# Botulinum Neurotoxin Injection for the Treatment of Recurrent Temporomandibular Joint Dislocation with and without Neurogenic Muscular Hyperactivity

**DOI:** 10.3390/toxins10050174

**Published:** 2018-04-25

**Authors:** Kazuya Yoshida

**Affiliations:** Department of Oral and Maxillofacial Surgery, National Hospital Organization, Kyoto Medical Center, 1-1 Mukaihata-cho, Fukakusa, Fushimi-ku, Kyoto 612-8555, Japan; kayoshid@kyotolan.hosp.go.jp; Tel.: +81-75-641-9161; Fax: +81-75-643-4325

**Keywords:** botulinum neurotoxin, temporomandibular joint dislocation, lateral pterygoid muscle, botulinum toxin therapy

## Abstract

The aim of this study was to compare treatment outcomes following intramuscular injection of botulinum neurotoxin (BoNT) in patients with recurrent temporomandibular joint dislocation, with and without muscle hyperactivity due to neurological diseases. Thirty-two patients (19 women and 13 men, mean age: 62.3 years) with recurrent temporomandibular joint dislocation were divided into two groups: neurogenic (8 women and 12 men) and habitual (11 women and 1 man). The neurogenic group included patients having neurological disorders, such as Parkinson’s disease or oromandibular dystonia, that are accompanied by muscle hyperactivity. BoNT was administered via intraoral injection to the inferior head of the lateral pterygoid muscle. In total, BoNT injection was administered 102 times (mean 3.2 times/patient). The mean follow-up duration was 29.5 months. The neurogenic group was significantly (*p* < 0.001) younger (47.3 years) than the habitual group (84.8 years) and required significantly (*p* < 0.01) more injections (4.1 versus 1.7 times) to achieve a positive outcome. No significant immediate or delayed complications occurred. Thus, intramuscular injection of BoNT into the lateral pterygoid muscle is an effective and safe treatment for habitual temporomandibular joint dislocation. More injections are required in cases of neurogenic temporomandibular joint dislocation than in those of habitual dislocation without muscle hyperactivity.

## 1. Introduction

Botulinum neurotoxin (BoNT) is produced by the gram-positive, anaerobic, spore-forming bacterium *Clostridium botulinum*, and is one of the most lethal biological toxins known to man. BoNT has seven antigenically different serotypes and exerts a paralytic action by rapidly and strongly binding to presynaptic cholinergic nerve terminals [1]. It is then internalized and ultimately inhibits the exocytosis of acetylcholine by decreasing the frequency of acetylcholine release. Without its nerve supply, the muscle fiber will deteriorate; however, the muscle will regain its strength as the nerves regenerate. The clinical applications of BoNT continue to expand in a variety of diseases such as strabismus, blepharospasm, cervical dystonia, Meige syndrome, spasmodic dysphonia, tics, tremor, and other movement disorders [2,3,4,5,6]. In the oral and maxillofacial region, BoNT has been used to treat oromandibular dystonia, hemifacial spasm, oral dyskinesia, synkinesis following defective healing of the facial nerve, temporomandibular disorders, bruxism, painful masseter hypertrophy, Frey’s syndrome, hypersalivation, trigeminal neuralgia, myofascial pain, and for aesthetic applications, such as perioral and other wrinkles [5,6,7,8].

The dislocation of the temporomandibular joint is defined as a non-reducing displacement of the condyle in front of, and superior to, the articular eminence. Dislocation of the temporomandibular joint is not rare and is observed relatively often in the field of oral and maxillofacial surgery. The dislocation is classified according to its course (acute, recurrent, or habitual), its direction (anterior, posterior, lateral, and superior), and the affected side (bilateral and unilateral) of the jaw. If acute dislocation becomes increasingly frequent and progressively aggravating, it is classified as recurrent or habitual dislocation. Recurrent temporomandibular joint dislocation can be seen in morphological changes such as atrophic eminence, temporomandibular joint laxity, occlusal disharmony, and functional changes such as neurogenic or drug-induced muscular hypertonus [9]. Dislocation can be anterior, posterior, lateral or superior. The most commonly occurring dislocation were anterior dislocations. Fracturing often accompanies dislocation in the posterior, lateral, or superior direction. The symptoms of bilateral dislocations, which are found commonly, include a fixed-open mouth, protruding mandible, pain in the masticatory muscles and temporomandibular joint, salivation, and difficulties with speech and eating. In cases of unilateral dislocation, the mandible exhibits deviation toward the contralateral side to the dislocated condyle. These symptoms are distressing and result in severe dysarthria, dysphasia, and masticatory disturbance, and require immediate treatment.

The lateral pterygoid, or the external pterygoid, is a muscle involved in mastication and usually has two heads: the inferior (lower) head and the superior (upper) head. The lateral pterygoid has a specialized role in the mouth opening that is mediated by horizontally oriented fibers of the inferior head. When the muscles of the bilateral inferior head contract together, the condyle is pulled forward and slightly downward. The mandible can be opened or protruded. If only one of the inferior head muscles contracts, the mandible rotates about a vertical axis, passing roughly through the opposite condyle and is pulled medially towards the contralateral side. Numerous methods have been reported for inserting electromyographic electrodes into the inferior and superior heads of the lateral pterygoid muscle [10,11,12,13,14,15,16,17,18,19,20,21,22,23,24,25,26]. Most of these methods involve “freehand” manual needle insertion, which occasionally results in complications such as hematoma or arterial bleeding [12,13] due to maxillary artery injury. Investigators have elucidated the course of the maxillary artery and found variation in this course between populations [27,28,29,30,31,32,33,34,35,36,37,38,39,40,41,42,43,44]. Although this variation is important during injection into the lateral pterygoid muscle, almost no attention has been paid to this anatomical difference.

In 1995, Daelen et al. [45] first described botulinum toxin injection into the lateral pterygoid muscle for the treatment of habitual temporomandibular joint dislocation. Several investigators subsequently reported using BoNT therapy for recurrent temporomandibular joint dislocation. They reported that it was effective and minimal unfavorable reactions were observed [46,47,48,49,50,51,52,53]. However, almost all previous studies have been single case reports or case series. As such, there is insufficient data regarding associated complications and the detailed application of the treatment.

In this report, differences in outcomes of BoNT therapy between patients with neurogenic temporomandibular joint dislocation and other habitual dislocations are examined. Additionally, the safety of injection into the inferior head of the lateral pterygoid muscle, with respect to the course of the maxillary artery, is discussed.

## 2. Results

The neurogenic group was significantly (*p* < 0.001, unpaired *t*-test) younger (47.3 ± 20.7 years) than the habitual group (84.8 ± 5.4 years). The percentage of female patients in the habitual group (91.7%) was significantly higher (*p* < 0.01, Fisher’s exact test) than the percentage in the neurogenic group (40%). The ratio of edentulous to dentate patients was significantly higher (*p* < 0.01, Fisher’s exact test) in the habitual group (66.7%) than in the neurogenic group (15%).

The results of the BoNT therapy for each patient are shown in Table 1. BoNT injections were administered 102 times in total (mean: 3.2 ± 2.8 times, range: 1–12 times). The dosage of BoNT per treatment session was 50.0 ± 6.4 units and 27.7 ± 7.6 units per side (Table 1). One injection was enough to prevent another episode in 9 of 32 (28.1%) patients from the habitual group. The percent of single injections was significantly higher (*p* < 0.01, Fisher’s exact test) in the habitual group (75%) than in the neurogenic group (0%). The neurogenic group required significantly (*p* < 0.01) more (4.1 ± 2.8 times) injections than the habitual group (1.7 ± 1.7 times). No significant immediate or delayed complications were observed. The mean follow-up period was 29.5 ± 19.9 months (range: 6–75 months) (Table 1).

## 3. Discussion

The present study is the first to report on differences in outcomes following BoNT injection into the inferior head of the lateral pterygoid muscle between patient groups presenting with neurogenic or habitual temporomandibular joint dislocation. Intramuscular injection of BoNT by the intraoral approach is very effective and causes no adverse reactions. A greater number of injections are required in cases of temporomandibular joint dislocation caused by neurological dysfunction than in those of dislocation presenting without muscle hypertonus.

### 3.1. Limitations

There are limitations in the interpretation of the data presented here. Significant differences in age, gender distribution, and edentulousness between two groups may bias the data and prevent adequate statistical analysis. However, these differences might represent the real characteristics of each type of dislocation. 

Referral bias is another possible limitation. The author produced a website for patients with involuntary movements of the stomatognathic region [54], and within the author’s department, a comprehensive range of multimodal therapies for involuntary movements of the orofacial region, including medication, muscle afferent block therapy [55,56,57], botulinum toxin therapy [25,26], splint therapy [58], and surgery [59,60] are offered. As the number of patients with involuntary movements presenting to the author’s department is high [61], temporomandibular joint dislocation derived from oromandibular dystonia constituted the majority of cases in this study. Therefore, the number of neurogenic temporomandibular joint dislocation and related diseases may be greatly influenced by the specialty of the investigator. Briefly, neurogenic patients may be more prevalent in the Department of Neurology, while habitual dislocation may be more common in a Department of Oral and Maxillofacial Surgery or Dentistry. 

Furthermore, in the current study, a control group was not included due to ethical reasons. Randomization and blinding were not included in this study. Despite ethical difficulties, in the future, evidence-based randomized control trials with a larger number of patients are required to further characterize the differences between neurogenic and simple habitual dislocation.

Radiographic examination was impossible due to the lack of compliance in patients with dementia, and also due to the involuntary movements associated with neurological diseases. Therefore, precise evaluations of temporomandibular joint dislocation, such as morphological characteristics or changes in the articular eminence, the condyle, and the temporomandibular joint disc, were lacking in this study. Additionally, dementia and involuntary movements impaired the investigation of symptoms associated with joint and muscle conditions, such as joint sounds, condylar movements, maximal mouth opening, and muscle tenderness. Although there were insufficient clinical findings in these areas, diagnosis based on the palpable dislocation of the condyle was simple and there were no issues with regard to the treatment of the dislocation. In future studies, a more precise description of symptoms, as well as radiographic examinations, are desirable.

### 3.2. Local Anatomy for Safe Injection

**Figure 1:**
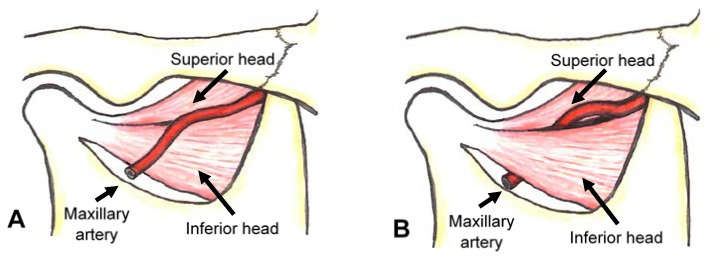
The lateral and medial courses of the maxillary artery to the lateral pterygoid muscle. The two main courses of the maxillary artery are lateral (**A**) and medial (**B**). In the lateral course, the maxillary artery passes lateral to the inferior head of the lateral pterygoid muscle (**A**). In the medial course, the artery passes medial to the muscle (**B**).

The maxillary artery, the larger terminal branch of the external carotid artery, arises behind the neck of the mandible and is initially embedded in the parotid gland. The maxillary artery passes lateral to the inferior head of the lateral pterygoid muscle or medial to the muscle (Figure 1).

**Figure 2:**
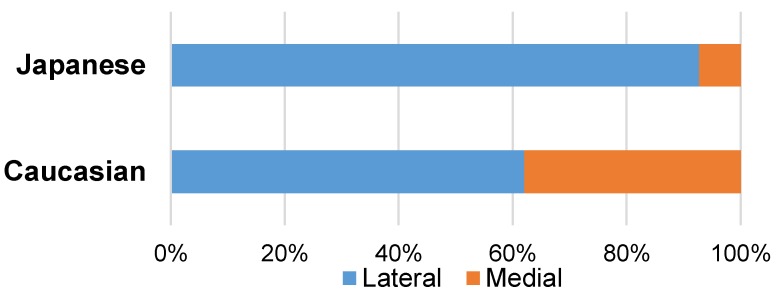
The frequency of the lateral and medial course of the maxillary artery in Japanese and Caucasian populations. The frequency was calculated based on the total data from previous studies in Japanese and Caucasian populations, which evaluated more than 100 cases [28,30,31,32,34,35,36,37,38,39,40,41,42,43]. The maxillary artery runs medially to the inferior head of the lateral pterygoid in 7.3% and 38% of Japanese and Caucasian individuals, respectively.

In 1928, Adachi [30] first reported a discrepancy in the frequency of the medial course of the maxillary artery to the inferior head of the lateral pterygoid muscle between Japanese and Caucasian populations. In 92.7% of Japanese subjects (and possibly other Asian groups), the maxillary artery passes lateral to the inferior head of the lateral pterygoid muscle (Figure 2). However, in Caucasian subjects, the artery passes medial to the muscle in a greater proportion of individuals (38%) (Figure 2).

Operators should be aware of these differences prior to performing the procedure as there is a decreased risk of injury to the maxillary artery is if the needle is inserted only once. However, with increased needle insertion, the risk of complications such as bleeding, hematoma, and swelling increases [26]. These are related to the injury of the maxillary artery. Although most reports on BoNT injection into the lateral pterygoid muscles used the extraoral preauricular transcutaneous approach for Caucasian patients, clinicians should consider the course of the maxillary artery to minimize complication related to injuries to the artery. 

### 3.3. Injection Methods into the Lateral Pterygoid Muscle

**Figure 3:**
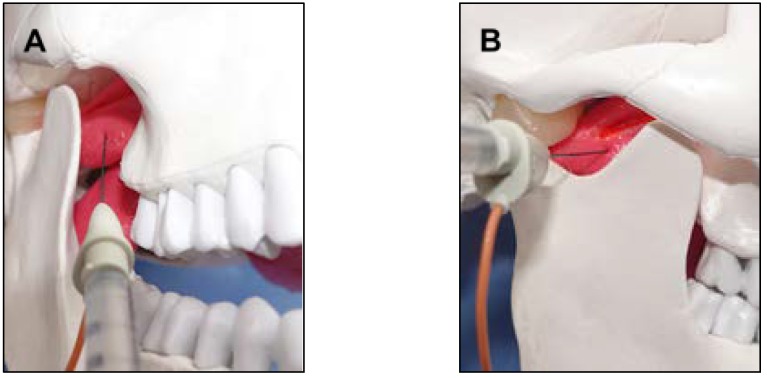
The intraoral (**A**) and extraoral (**B**) approaches for lateral pterygoid muscle injection.

The inferior head of the lateral pterygoid muscle can be accessed via intraoral and extraoral transcutaneous routes (Figure 3). Various methods used for inserting electromyographic electrodes into the inferior and superior heads of the lateral pterygoid muscle have been reported [10,11,12,13,14,15,16,17,18,19,20,21,22,23,24,25,26]. However, the intraoral approach is preferable for several reasons [15,25,26]. First, this approach leads to less patient anxiety as it is similar to the approach employed during routine intraoral injections in dental treatment. Second, it reduces the risk of damage to the maxillary artery, and third, during the extraoral preauricular transcutaneous approach, the needle electrode could be bent or broken if the patient bites down forcefully. 

As previously stated, the greater the number of times the needle is inserted, the greater the risk of complications and pain [15,25]. Thus, the more accurately the needle is inserted, the more likely the improvement in the patient’s condition is, and the less likely complications are. BoNT therapy for oromandibular dystonia is more effective when it is administered to the jaw closing muscles than when it is administered to the lateral pterygoid muscle [62], which may reflect the anatomical complexity of the lateral pterygoid muscle and the resulting difficulty of accurate needle insertion [25,26]. 

**Figure 4:**
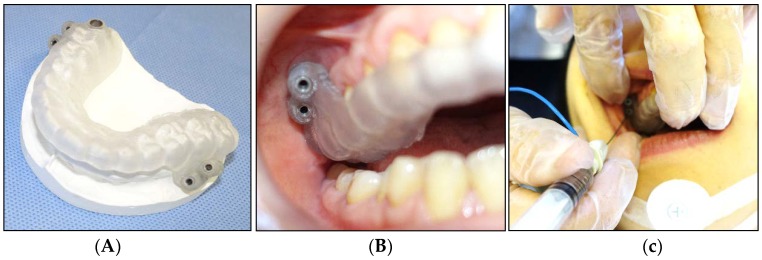
The needle guide using computer-aided design/computer-assisted manufacture-based data. Two points at the center of the inferior head of the lateral pterygoid muscle are selected, after checking the orientation and volume of the lateral pterygoid muscles on computed tomography [26]. A needle guide is fabricated using the stereolithographic method (**A**). The guide is inserted into the oral cavity and stabilized with the help of the teeth (**B**). A disposable hypodermic needle electrode is inserted through the metal sleeves (**C**).

Dystonia is a movement disorder that is characterized by sustained or intermittent muscle contractions that cause abnormal, often repetitive, movements, postures, or both [63]. Oromandibular dystonia is a focal type of dystonia involving the masticatory and/or lingual muscles [58,59,64,65]. Patients with oromandibular dystonia often exhibit dystonic contracture of the lateral pterygoid muscle [19,20,21,22,23,25,26,53,55]. Injection of BoNT into the lateral pterygoid muscle requires skill, sufficient anatomical knowledge, and experience, and such injections are rarely administered; therefore, it is difficult, practically speaking, to become skilled in this procedure [25,26]. Since 1988, the author has performed this procedure several thousand times for electromyographic studies [14,15,16,17], muscle afferent block [19,54,56,57], and BoNT therapy [25,26,53,54,64]. Recently, a method using a computer-aided design/computer-assisted manufacture-derived needle guide (Figure 4) was published [26]. The use of the needle guide allows clinicians to perform the injection procedures without any complications at skill levels that would normally require several years to achieve. The needle insertion guide has enabled development of what the author considers to be the most accurate and safe method for injecting BoNT into the inferior head of the lateral pterygoid muscle reported so far [26].

It is very difficult to accurately and safely inject BoNT using the intraoral approach in patients with an extremely narrow space between the coronoid process and maxilla [25,26]. Indeed, despite repeated BoNT injections using the intraoral approach, there can be decreased beneficial effects. This might be related to the fact that BoNT is injected into a limited part of the muscle. For such patients, the extraoral preauricular percutaneous approach is an alternative option. After careful palpation of the infratemporal fossa, clinicians can insert the needle vertically into the skin [25]. The needle should be inserted 20–25 mm through the mandibular notch. After aspiration, BoNT is injected into the muscle. The correct placement of the needle is verified by evaluating the electromyographic burst during mouth opening or protrusion. If patients can open their mouth according to the clinician’s instruction, confirmation of correct needle placement is easy. However, patients with severe mental impairment have to be injected under general anesthesia. In such cases, the correct placement can be verified by eliciting jaw reflex [48] or by the use of a nerve stimulator [51].

Excluding adverse effects related to injuries to the maxillary artery or the pterygoid nervous plexus, previously reported complications, which postulated diffusion of the BoNT into adjacent muscles, include transient dysphagia, painful chewing, dysarthria, nasal regurgitation, or nasal speech [66]. However, all complications were reported to subside within 2–4 weeks [66]. In this study, BoNT injections were administered more than 100 times via the intraoral route, and no immediate or delayed adverse reactions were detected.

### 3.4. BoNT Therapy for Recurrent Temporomandibular Joint Dislocation

Acute temporomandibular joint dislocation can be reduced using Hippocratic maneuvers: specifically, applying downward pressure on the posterior teeth and upward pressure on the chin while pushing the mandible posteriorly. However, in some cases, this requires sedation or general anesthesia. Conservative treatments of temporomandibular joint dislocation include physical therapy, chin cap, muscle relaxants, splint therapy, and instruction to avoid wide mouth opening [9]. Various surgical interventions are also used to treat this condition. These procedures include augmentation of the height of the articular eminence, reduction of the height of the eminence, intermaxillary fixation, and the injection of sclerosant or autologous blood around the temporomandibular joint [9,67]. 

To the best of our knowledge, the first BoNT injection for the purpose of treating habitual temporomandibular joint dislocation was described in 1995 by Daelen et al. [45]. They were also the first to propose the term “neurogenic temporomandibular joint dislocation”, which was derived from neurological diseases such as multiple sclerosis or Parkinson’s disease [46]. Following this study, several investigators reported using BoNT therapy for recurrent temporomandibular joint dislocation [45,46,47,48,49,50,51,52,53]. All these investigators reported that this treatment was effective and had minimal adverse effects. 

The proper dosage of BoNT for the successful treatment of temporomandibular joint dislocation remains unclear. Although the dosage is empirical, the lowest sufficiently effective dose is the best. The frequent administration of BoNT in high doses could result in the development of antibodies. The author normally uses a 50-unit vial (Botox^®^). If the dislocation occurred unilaterally, 50 units were injected into the inferior head of the lateral pterygoid on the dislocated side (Table 1). If dislocation occurred bilaterally, 25 units were administered into the muscles on each side (Table 1). For patients with oromandibular dystonia, the dose of BoNT is determined according to the volume of the target muscles and the strength of the muscle contraction [25,26]. Further studies with larger samples are necessary to determine the optimal treatment dosage.

### 3.5. Differences in the Pathophysiology between Neurogenic and Habitual Dislocation

There are many causes of dislocation, including trauma, excessive mouth opening during yawning, dental or otorhinolaryngological examinations or treatments, vomiting, hypermobile or deranged temporomandibular joints, and involuntary movements [9]. Furthermore, dislocation can be influenced by other conditions such as neurogenic muscle hyperactivity, pathological osseous conditions affecting the articular eminence, and connective tissue disorders [9]. Therefore, it is likely that the pathophysiology of neurogenic and habitual temporomandibular joint dislocations is different. In this study, the diseases present in the neurogenic group included dystonia, Parkinson’s disease, and progressive supra atrophy. One patient with oromandibular dystonia exhibited temporomandibular joint dislocation due to excessively strong muscle contraction in the lateral pterygoid muscle that subsequently resulted in the complete obstruction of the patient’s upper airway [53]. For such patients, a higher dosage of BoNT and repeated injections are necessary to reduce the extraordinary muscle contraction. In these patients, the lateral pterygoid muscle showed excessively forceful involuntary contracture. Therefore, the BoNT injection must be repeated significantly more times than for the habitual group.

In elderly patients, temporomandibular joints are easily dislocated due to the atrophy of the articular eminence, joint laxity, and edentulousness. The percentage of edentulous patients was significantly higher in the habitual group than in the neurogenic group, in this study. A previous study [50] assumed that the cause of dislocation was a muscular imbalance between the jaw opening and jaw closing muscles caused by neuromuscular dysfunction. These patients rarely have hyperactive lateral pterygoids and BoNT therapy can be applied. In the habitual group, it is not forceful involuntary movements, but a disharmony between the jaw opening and jaw closing muscles, and also an atrophic articular eminence, that may result in dislocation. Although this can be treated by an eminectomy, this intervention may be ineffective due to poor health. The effects of BoNT diminishes over time; however, BoNT therapy can prevent another dislocation of the temporomandibular joint for a few months, and fibrosis around the temporomandibular joint may make another occurrence less likely. 

In the past, if conservative treatment was not successful, surgical intervention was the only option [9,67]. The method described in this study should be the first choice treatment for temporomandibular joint dislocation, particularly for elderly patients who cannot tolerate conservative methods and are vulnerable to complications associated with operative procedures. If the cause of the temporomandibular joint dislocation is morphological, surgical procedures can be helpful. However, neurogenic hyperactivity of the inferior lateral pterygoid muscle cannot be relieved by surgical intervention, and BoNT injection into the inferior head of the lateral pterygoid muscle is helpful for such patients. Many clinicians have begun to recognize this therapy as an attractive option. Recently, the number of patients being referred for this treatment has been increasing rapidly. 

## 4. Conclusions

The intramuscular injection of BoNT into the inferior head of the lateral pterygoid muscle via the intraoral route is highly effective and safe for treating patients with recurrent temporomandibular joint dislocation. This method should be the first choice in patients for whom surgical procedures are contraindicated.

## 5. Materials and Methods 

### 5.1. Patients

Thirty-two patients with recurrent temporomandibular joint dislocation (19 women and 13 men; mean age with standard deviation [SD]: 62.3 ± 24.0 years), all of whom were intolerant to conservative methods, visited the author’s department from July 2007 to June 2017. All the patients, or their legal guardians, provided written informed consent after listening to a detailed and complete explanation of the planned treatment. This study was performed in accordance with the Declaration of Helsinki after obtaining the approval of the institutional review board and ethics committee of the Kyoto Medical Center. The demographic characteristics of the patients are summarized in Table 2. 

The diagnosis of recurrent temporomandibular joint dislocation was based on the past history and current symptoms, such as the inability to close the mouth in the intercuspal position due to an open-locked position and the palpable dislocation of the condyle from the fossa. Twenty-eight patients had bilateral, and four had unilateral, temporomandibular joint dislocation (Table 2). Although radiographic examination was impossible in most cases with dementia due to the lack of patient compliance, the diagnosis was made easily based on symptoms. Patients were divided into two groups; neurogenic (8 women and 12 men; mean age: 47.3 ± 20.7 years) and habitual (10 women and 1 man; mean age: 84.8 ± 5.4 years) (Table 2). The neurogenic group included patients with neurological disorders that are accompanied by muscular hypertonus. Muscle hyperactivity was confirmed by electromyographic examination, or from the tenderness or hardness during palpation of the lateral pterygoid muscle. The diseases present in the neurogenic group included oromandibular dystonia (20 patients), Parkinson’s disease (3 patients), corticobasal degeneration (1 patient), multiple system atrophy (1 patient), and progressive supranuclear palsy (1 patient) (Table 2). The author diagnosed oromandibular dystonia based on the patients’ electromyographic findings and the characteristic clinical features such as stereotypy, task-specificity, co-contraction, and morning benefit [55,58,59,64]. Other neurological diseases were diagnosed previously by qualified neurologists. Patients who had no neurological diseases accompanying muscle hyperactivity were classified as the habitual group. Ten of the twelve (83.3%) patients in the habitual group had dementia. Ten of the thirty-two (31.3%) patients were currently being prescribed psychiatric medication and had tardive dystonia. Twelve patients (37.5%) had other dystonia in a different part of their body, including cervical dystonia (7 patients), writer’s cramp (3 patients), generalized dystonia (3 patients) and blepharospasm (1 patient) (Table 2). Eleven patients (34.4%) used full dentures or were edentulous. 

### 5.2. Botulinum Neurotoxin (BoNT) Therapy

BoNT type A (Botox^®^, Allergan, Irvine, CA, USA) was reconstituted with normal saline to reach a final concentration of 2.5–5 units/0.1 mL. This procedure was normally performed using a final concentration of 2.5 units/0.1 mL. If the injection was unilateral or required a 100-units vial (Botox^®^), BoNT was reconstituted to 5 units/0.1 mL. The insertion point was the mucobuccal fold of the distal root of the upper second molar. After a gargle with a 50-fold diluted solution of Neostelin Green 0.2% mouthwash solution (Nippon Shika Yakuhin, Yamaguchi, Japan), a disposable hypodermic needle electrode (TECA™ MyoJect™ Luer Lock, 37 mm × 25 G, Natus Neurology Incorporated, Pleasanton, CA, USA) was angled posteriorly and superiorly by 30 degrees in relation to the occlusal plane, and medially by 20 degrees [25,26]. The needle was inserted to a depth of 20–30 mm without local anesthesia. After aspiration that the needle had not pierced a blood vessel, 25–50 units of BoNT were injected into the muscle. The correct placement of the electrode was verified by evaluating the full recruitment of electromyographic signal during mouth opening, or contralateral mandibular movement using an electromyographic apparatus (Neuropack n1, MEM-8301, Nihon Kohden, Tokyo, Japan), after amplification and filtering (low-cut filter, 10 Hz; high-cut filter, 3 kHz), and digitized with a sampling frequency of 10 kHz and 16-bit resolution [24]. The effects of BoNT manifested anywhere from 2–3 days to 1–2 weeks following treatment. Therefore, patients were vulnerable to another episode of dislocation during this time. Patients were carefully observed during this period. The injections were repeated over time if the patients showed involuntary mouth opening or experienced another dislocation. If the first injection was not sufficiently beneficial, a second injection was administered after 2 months. Successive injections must be administered, at minimum, every 3 months. Patient follow-up continued until no dislocation occurred for at least 6 months. All procedures were conducted under outpatient conditions without sedation or general anesthesia. For ethical reasons, a control group was not used in this study.

### 5.3. Statistical Analysis

The demographic characteristics and the results of BoNT therapy were compared between the neurogenic and habitual groups. An unpaired *t*-test was used to compare the data obtained from the two groups. Fisher’s exact test was used to assess the statistical significance of differences in the distributions between the two groups. All analyses were performed using SPSS version 14.0 (SPSS Japan Inc., Tokyo, Japan). A value of *p* < 0.05 was considered statistically significant.

## Figures and Tables

**Table 1:** The results of the treatment of the BoNT injection.

Patient No.	Group	Dosage per Time (Units)	Dosage per Side (Units)	Botox Injection (Times)	Follow-Up (Months)
1	N	25	25	2	12
2	N	50	25	2	6
3	N	50	25	5	36
4	N	50	25	8	38
5	N	50	25	12	48
6	N	50	25	2	12
7	N	50	25	6	28
8	N	50	25	2	29
9	N	50	25	8	52
10	N	50	25	2	45
11	N	50	50	3	51
12	N	50	25	4	59
13	N	50	25	8	75
14	N	50	25	2	26
15	N	50	25	2	15
16	N	50	25	2	13
17	N	50	25	5	31
18	N	50	25	2	19
19	N	50	25	3	10
20	N	75	37.5	2	15
21	H	50	50	1	27
22	H	50	25	7	56
23	H	50	25	1	6
24	H	50	25	1	9
25	H	50	50	1	10
26	H	50	25	1	9
27	H	50	25	2	12
28	H	50	25	1	47
29	H	50	25	1	53
30	H	50	25	2	15
31	H	50	25	1	14
32	H	50	25	1	65
Mean (SD)		50.0 (6.4)	27.7 (7.6)	3.2 (2.8)	29.5 (19.9)

N: neurogenic group, H: habitual group.

**Table 2:** The patients’ demographic characteristics.

No.	Group	Age (Years)	Sex	Side	Duration (Months)	Frequency (Times/Week)	Diseases Causing Muscle Hyperactivity	Other Diseases	Denture
1	N	33	F	Uni	30	1	OMD, CD	schizophrenia	-
2	N	86	F	Bi	8	21	corticobasal degeneration, OMD	dementia, HT	+
3	N	43	M	Bi	36	3	OMD, CD, blepharospasm	depression	-
4	N	38	M	Bi	180	0.5	PD, generalized dystonia	-	-
5	N	30	M	Bi	8	1	OMD, CD	schizophrenia	-
6	N	53	M	Bi	24	2	OMD, WC	-	-
7	N	51	F	Bi	1	3	OMD, WC, CD	-	-
8	N	48	M	Bi	120	1	PD, OMD, CD	sleep apnea syndrome	-
9	N	66	M	Bi	120	2	PD, OMD	depression	-
10	N	35	F	Bi	36	7	OMD	schizophrenia	-
11	N	50	M	Uni	6	3	OMD	scoliosis	-
12	N	67	F	Bi	36	7	generalized dystonia	-	-
13	N	29	F	Bi	12	5	OMD	depression	-
14	N	35	M	Bi	10	10	OMD	panic disorder	-
15	N	19	F	Bi	6	3	OMD, CD, WC	depression	-
16	N	42	M	Bi	1	14	OMD, CD	dementia	-
17	N	21	M	Bi	60	7	generalized dystonia	hypoxia, DM	-
18	N	84	F	Bi	8	14	OMD	dementia, HT	+
19	N	64	M	Bi	6	21	multiple system atrophy, OMD	-	-
20	N	80	M	Bi	5	2	progressive supranuclear palsy, OMD	-	+
21	H	79	F	Bi	3	2	-	dementia, CI, HT	-
22	H	87	F	Uni	120	0.5	-	CI, HT, heart failure	+
23	H	87	F	Bi	1	1	-	dementia, osteoporosis, pneumonia	+
24	H	84	F	Bi	6	0.5	-	dementia	+
25	H	98	F	Uni	3	14	-	HT	-
26	H	86	F	Bi	8	21	-	dementia, HT, gastric ulcer	+
27	H	84	M	Bi	2	23	-	dementia	+
28	H	80	F	Bi	6	14	-	dementia	+
29	H	88	F	Bi	84	7	-	dementia, heart failure, breast cancer, depression	-
30	H	83	F	Bi	3	7	-	dementia, HT, pneumonia	+
31	H	85	F	Bi	6	2	-	dementia, HT	+
32	H	77	F	Bi	4	14	-	dementia, HT, CI, cervical spondylosis	-
Mean (SD)		62.3 (24.0)	-		30.0 (45.3)	6.7 (6.5)	-	-	

N: neurogenic group, H: habitual group, Bi: bilateral, Uni: unilateral, OMD: oromandibular dystonia, CD: cervical dystonia, WC: writer’s cramp, PD: Parkinson’s disease, HT: hypertension, DM: diabetes mellitus, CI: cerebral infarct.
